# The overlap of ANCA associated vasculitis with Sjögren’s syndrome in elderly male patient: a case report and literature review

**DOI:** 10.3389/fmed.2026.1679800

**Published:** 2026-02-24

**Authors:** Mengting Zhu, Ruobing Zhu, Haoran Zhu, Wenze Jiang, Yulu Jia, Wo Zhang, Zhongjie Qu, Keda Lu

**Affiliations:** 1 Department of Nephrology, The Third Hospital Affiliated to Zhejiang Chinese Medical University, Hangzhou, China; 2 Tianjin Medical University, Tianjin, China

**Keywords:** ANCA-associated vasculitis, csae report, overlap syndrome, rituximab, Sjögren’s syndrome

## Abstract

Anti-neutrophil cytoplasmic antibody (ANCA) associated vasculitis (AAV) is a rare disease characterized by inflammation of small vessels in various organs, usually affecting the kidneys, lungs, upper respiratory tract, skin, eyes, and peripheral nerves. The typical renal pathological manifestation is necrotizing crescentic glomerulonephritis. Sjögren’s syndrome is another relatively uncommon autoimmune disorder characterized by dry eyes and dry mouth. Here, we present a case of overlap syndrome involving AAV with SS in an elderly male patient who demonstrated significant improvement following aggressive treatment with cyclophosphamide, glucocorticoids, hemodialysis, and rituximab. In addition, we also found 49 cases of overlapping syndrome of ANCA combined with SS through literature review and summarized and analyzed them.

## 1 Introduction

Anti-neutrophil cytoplasmic antibody (ANCA)-associated vasculitis (AAV) constitutes a spectrum of systemic vasculitides characterized by the ANCA presence, primarily affecting small and medium-sized vessels (1–3). This category encompasses three clinicopathological entities: granulomatosis with polyangiitis (GPA, formerly known as Wegener’s granulomatosis), microscopic polyangiitis (MPA), and eosinophilic granulomatosis with polyangiitis (EGPA, formerly known as Churg-Strauss). The incidence rate of MPA is 0.0013% (4, 5). Sjögren syndrome (SS) is a chronic systemic autoimmune disorder typified by lymphocytic proliferation and progressive damage to exocrine gland. The prevalence of SS ranges from 0.01% to 0.72%, with a notable predilection for females (male-to-female ratio approaching 10:1), while occurrences in children and the elderly are infrequent (6).

Here we report a case of an elderly male patient with overlap syndrome involving SS and AAV, who initially presented with renal insufficiency and tested positive for multiple specific autoantibodies. Renal pathology showed focal segmental necrotizing glomerulonephritis (FSNGN) with pauci-immune complex and chronic interstitial nephritis. After receiving cyclophosphamide (CTX), steroid pulse therapy, and low-dose rituximab (RTX), along with hemodialysis for renal support, the patient achieved clinical remission.

## 2 Case report

A 65-year-old man presented to our hospital in June 2021 complaining of xerostomia, ocular dryness, and fatigue. Throughout this period, his serum creatinine levels had been consistently around 110 μmol/L. However, 1 week prior to admission, there was a gradual increase in serum creatinine levels, rising from 108 to 165 μmol/L. Urinalysis revealed the presence of proteinuria and 165 red blood cells in the urine. Physical examination revealed diffuse erythema on the face, chest, and abdomen, with raised lesions that blanched upon pressure. Additionally, Raynaud’s phenomenon was noted in both hands. The results of the remaining physical examination were normal. In addition, he presented with a 2-year history of gout, a 10-year history of hypertension, and a 5-year history of coronary atherosclerotic heart disease, for which he had been receiving long-term treatment with metoprolol, losartan potassium, aspirin, and rosuvastatin. However, there was no familial predisposition to renal or rheumatic diseases.

**FIGURE 1:**
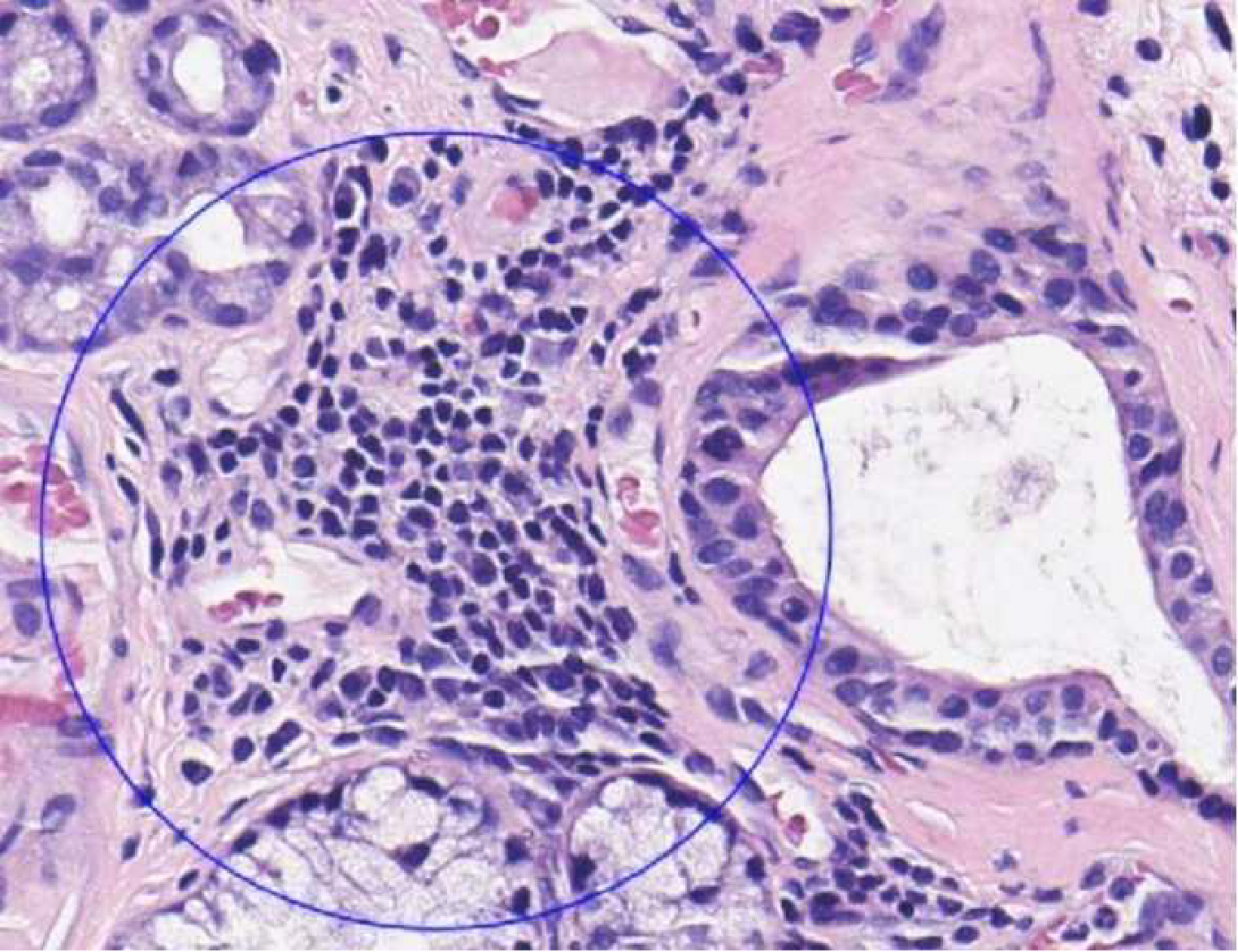
Biopsy of the labial gland showed infiltration of lymphocytes and plasma cells.

Upon admission, the patient presented with a temperature of 36.7 °C, a pulse rate of 66 beats per minute, and a blood pressure of 144/56 mmHg. Urinalysis revealed proteinuria and the presence of 165 red blood cells/μl (reference range: 0—15/μl) and a 24-h urinary protein level of 341 mg/24 h (reference range: <150 mg/24 h). Hematological parameters indicated white blood cell count (WBC) of 5.71 × 10^9^/L (reference range: 3.50–9.50 × 10^9^/L), hemoglobin of 109 g/L (reference range: 130–175 g/L), and blood platelets (PLT) of 119 × 10^9^/L (reference range: 125–350 × 10^9^/L), along with a serum creatinine level of 219.0 μmol/L (reference range: 57.0–111.0 μmol/L) and uric acid level of 438 μmol/L (reference range: 208–428 μmol/L). Immunological investigations revealed positive antinuclear antibody IgG (titer 1:320), anti-SS-A antibody, and markedly elevated anti-Ro-52 antibody, with additional positive results for anti-double-stranded DNA (dsDNA) antibody and perinuclear-ANCA (p-ANCA), while anti-myeloperoxidase (MPO) antibody was also positive, and cytoplasmic-ANCA (c-ANCA) and anti-proteinase 3 (PR3) antibody were negative. Serum immunoglobulin levels were elevated, with IgG at 41.19 g/L (reference range: 8.60–17.40 g/L), Ig KAPPA level at 5.46 g/L (reference range: 1.7–3.7 g/L), Ig LAMBDA level at 2.54 g/L (reference range: 0.9–2.1 g/L), the Ig KAPPA to Ig LAMBDA ratio is 2.15 (reference range: 0.26–1.65), and IgA level at 5.61 g/L (reference range: 1–4.2 g/L). Normal findings were observed for serum complement factors C3, C4, IgM, anti-glomerular basement membrane (GBM) antibody, antiphospholipid antibody profile, lymphocyte subsets, and rheumatoid factor. Serological tests for hepatitis B virus surface antigen, hepatitis C virus antibody, human immunodeficiency virus antibody, and cryoglobulins were negative. Electrocardiography revealed a normal sinus rhythm. Chest CT scan demonstrated bilateral ground-glass opacities and pulmonary cysts in both lungs. Parotid ultrasound revealed bilateral small-sized parotid glands. Salivary gland biopsy exhibited lymphocyte and plasma cell infiltration with four lesions per 10 mm^2^ (Figure 1).

**FIGURE 2:**
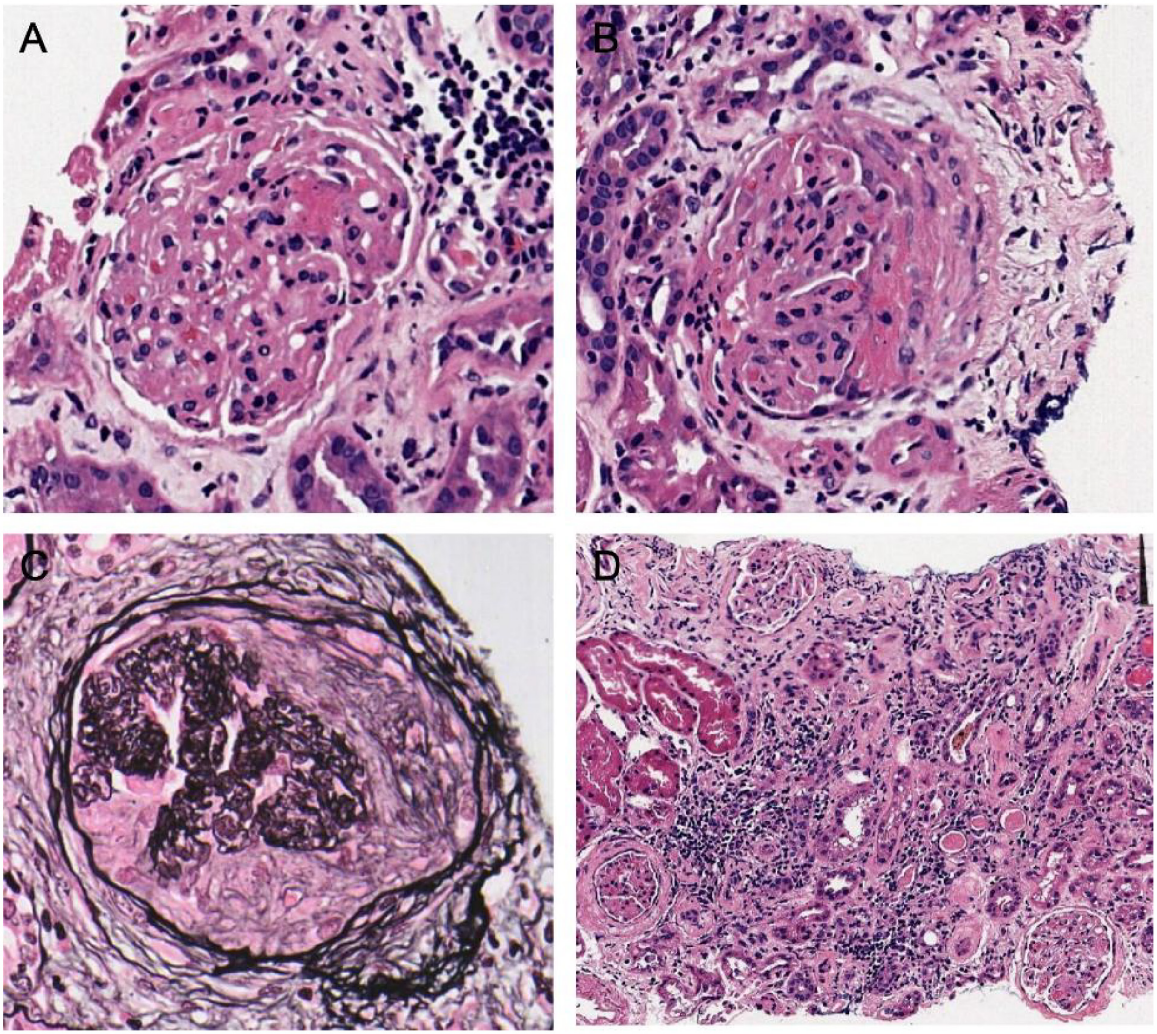
The pathologic features of renal biopsy. **(A)** Necrotizing crescents of the glomeruli and the capillary loops exhibited fibrinoid necrosis were observed with Hematoxylin-eosin (HE) staining in light microscopy (400×). **(B)** The presence of cellular crescent formation and peribulbar fibrosis was observed (HE 400×). **(C)** Periodic acid-silver methenamine (PASM) staining showed chronic crescentic formation, peribulbar fibrosis of the glomeruli, and ischemic shrinkage of the capillary loops in the glomeruli (400×). **(D)** Renal interstitial inflammation and multiple foci of small lymphocyte aggregation were observed (HE 200×).

**FIGURE 3:**
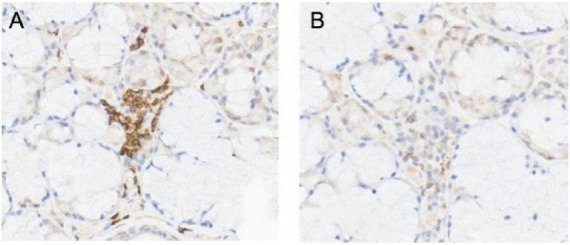
Immunohistochemistry showed multifocal T cell (CD3)+ **(A)**/focal B cell (CD20)+ **(B)**.

Renal pathology (Figure 2): A total of 25 glomeruli were observed by microscopy, among which seven exhibited glomerulosclerosis. The remaining glomeruli show fibrinous necrosis in individual capillary sinuses, along with the presence of five cellular crescents, one fibrous crescent, and one microcellular crescent. Mild proliferation of mesangial cells and matrix was observed. Multifocal and patchy atrophy (approximately 60% atrophy area) as well as inflammatory cell infiltration (mainly lymphocytes) with associated fibrosis were evident within the renal interstitium. Immunofluorescence microscopy did not detect depositions of IgG, IgM, IgA, C3, C1q, amyloid A, and light chains. Immunohistochemical staining indicated weak MPO positivity, along GBM and in measngium area with a higher proportion of T cells compared to B cells within the infiltrated lymphocyte areas (Figure 3). Electron microscopy revealed mild proliferation in the mesangial area along with a small amount of electron-dense deposits (EDD). Segmental thickening ranging from approximately 250–560 nm was observed in the basement membrane alongside segmental shrinkage. Occasional coarse granular EDD (primarily located at the mesangial waist) could be seen beneath the epithelium. Partial fusion of foot processes was also identified. Overall findings are consistent with AAV, and immune complex-mediated nephritis was suspected to be associated with SS.

The patient satisfied the American College of Rheumatology/European Alliance of Associations for Rheumatology (ACR/EULAR) classification criteria for AAV specifically MPA and SS (7, 8).

After the diagnosis was confirmed, the patient began to receive steroid pulse therapy (methylprednisolone 80 mg qd for 5 days) combined with CTX 400 mg (6.67 mg/kg) for immunosuppressive therapy. CTX was administered on 30 June 2021, and 7 July 2021. The steroid dose was adjusted several times according to the changes of the patient’s condition. However, despite treatment, the patient’s serum creatinine levels continued to fluctuate and rise. By 18 July 2021, the serum creatinine had increased to 465.8 μmol/L, prompting the initiation of hemodialysis twice a week starting on 21 July 2021.

Prior to the third CTX injection, the patient experienced progressive declines in PLT and WBC counts, with PLT reaching a minimum of 37 × 10^9^/L and WBC reaching a minimum of 1.34 × 10^9^/L. Bone marrow biopsy showed mature trilineage differentiation without significant abnormalities. After consultation with the hematology department, the patient was diagnosed with secondary immune-related thrombocytopenic purpura (ITP). CTX was stopped and steroid pulse therapy, intravenous immunoglobulin (IVIG) 20 g qd for 5 days, human granulocyte stimulating factor, recombinant human thrombopoietin, plasma and platelet transfusion were used. On 13 August 2021, the patient stopped hemodialysis and was converted to oral methylprednisolone. After adjusting treatment due to low fever, his body temperature returned to normal. In mid-September, the 24-h urine protein was 402.30 mg, serum creatinine was 354.6 μmol/L, antinuclear antibody IgG, anti-SSA antibody, anti-Ro-52 antibody, and p-ANCA were positive, and anti-PR3 antibody and anti-MPO antibody were negative. Chest CT showed a cavity in the upper lobe of the right lung. Through fungal culture and lung CT scans, we diagnosed the patient with aspergillosis pneumonia. Treatment with voriconazole tablets was administered (22 September 2021– 19 November 2021). Post-treatment lung CT scans revealed a reduction in the size of the cavities compared to previous imaging. According to the KDIGO guidelines, immunotherapy with RTX is used to achieve B-cell CD19 depletion, followed by hydroxychloroquine treatment for Sjögren’s syndrome (SS).

**FIGURE 4:**
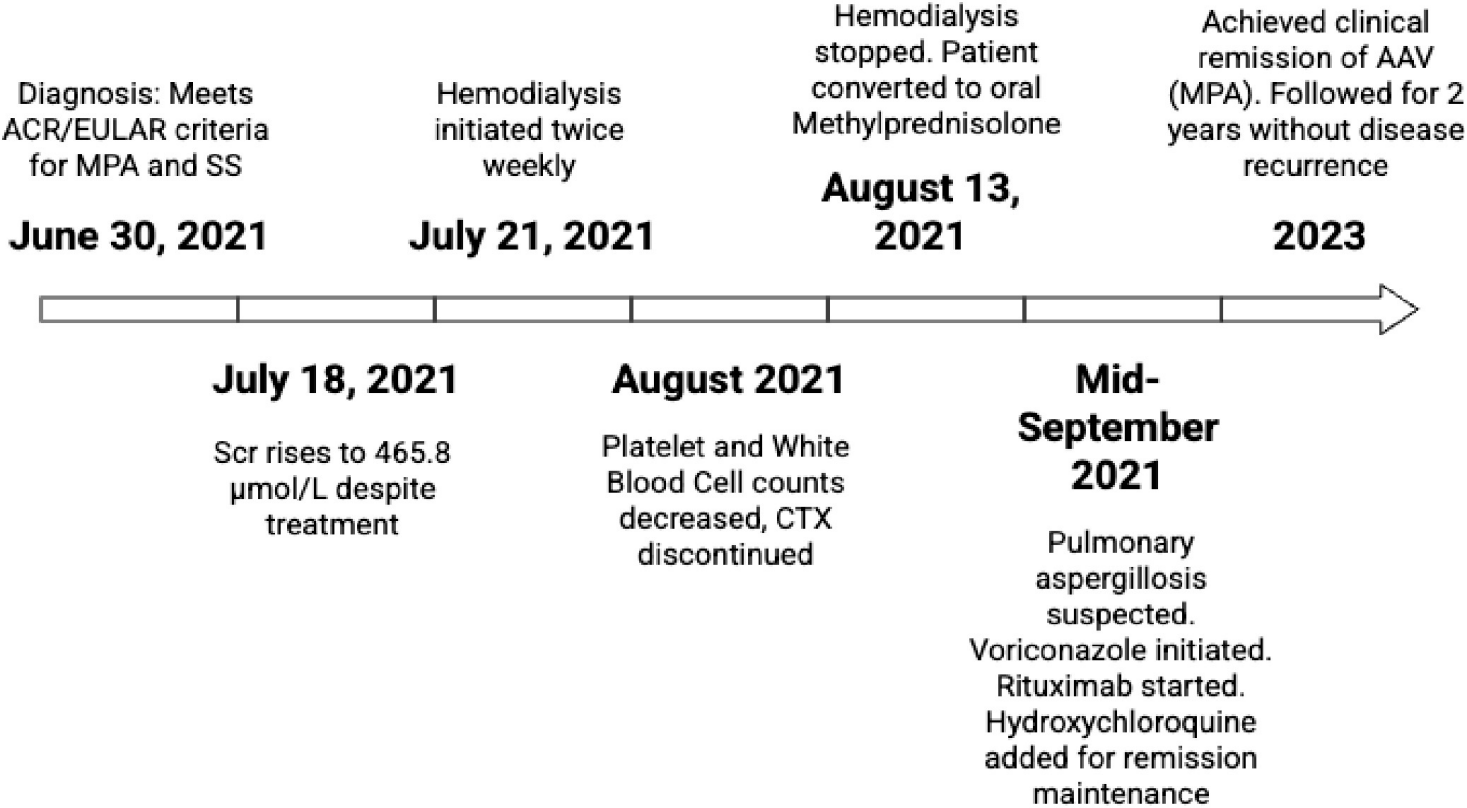
Timeline.

Since then, methylprednisolone and hydroxychloroquine have been continued, and despite dry mouth and eyes, the serum creatinine has stabilized at approximately 240 μmol/L, urinary protein has been present, and urinary red cells are absent. Anti-SSA antibody and anti-Ro-52 antibody were positive, while c-ANCA, p-ANCA, anti-PR3 antibody, anti-MPO antibody and dsDNA antibody were negative. The patient had achieved clinical remission of AAV and was followed up for 2 years without disease recurrence (Figure 4).

## 3 Literature review

Methods: Information related to cases reported to date has been collected by a systematic review of the literature. We conducted a search in Medline, Embase, Web of science, and Cochrane Library, from their inception to July 2024. We used the following MeSH terms on PubMed (“Antineutrophil cytoplasm antibody” OR “ANCA-associated vasculitis” OR “granulomatosis with polyangiitis” OR “Wegener” OR “microscopic polyangiitis” OR “eosinophilic granulomatosis with polyangiitis” OR “Churg-Strauss” OR “polyangiitis”) AND (“Sjögren’s syndrome”), and their equivalent on the other databases. There was no language neither time restriction.

Our literature search identified a total of 3,579 articles in the selected databases. No relevant articles were found in the gray literature. A total of 229 duplicate studies were removed. A total of 3,350 articles were screened: 75 articles were not retrieved for eligibility assessment. In the rest of the 3,275 articles, 3,237 articles were excluded from, because they do not conform to the diagnosis of AAV (*n* = 457), does not conform to the diagnosis of SS (*n* = 578) or does not include the case report (*n* = 2,202). Ultimately, 38 articles were included, involving 49 patients, along with a new patient at our center.

Statistical analysis: Quantitative variables were described as medians and interquartile ranges and compared with the Mann-Whitney test. Qualitative variables were described as percentages and compared with χ2 or Fischer’s exact test, as appropriate. All statistical analyses were performed using SPSS27. Statistical differences were considered significant when *p* < 0.05 (Table 1).

**TABLE 1:** Demographic and characteristics of 49 patients with SS and AAV, according to the subtypes of AAV.

General situation	MPA (*n* = 38)	GPA (*n* = 8)	EGPA (*n* = 2)	Total (*n* = 49)	*P*
Age, years [IQR]	61.5 [51.75–70.25]	63.4 [58.5–68]	54.5	61.5 [55.5–69.5]	/
Female, *n* (%)	31 (81.6)	8 (100)	1 (50)	40 (81.6)	/
Interval time, months	51.9	11.75	0	43.1	/
**SS features, *n* (%)**					
-Sicca syndrome	37 (97.4)	8 (100)	2 (100)	48 (98)	0.874
-Cutaneous	15 (39.5)	2 (25)	0	17 (34.7)	0.417
-Musculoskeletal system	15 (39.5)	2 (25)	0	17 (34.7)	0.417
-Respiratory system	19 (50)	7 (87.5)	2 (100)	29 (59.2)	0.07
-Digestive system	5 (13.2)	1 (12.5)	0	6 (12.2)	0.86
-Renal	34 (89.5)	1 (12.5)	0	36 (73.5)	/
-Nervous system	9 (23.7)	2 (25)	1 (50)	12 (24.5)	0.704
-Hematological system	15 (39.5)	2 (25)	0	17 (34.7)	0.417
-Cryoglobulinemia	2 (5.3)	1 (12.5)	0	3 (6.1)	0.694
-AITD	3 (7.9)	0	0	3 (6.1)	0.656
-anti-SSA/SSB antibodies positive	33 (86.8)	4 (50)	0	38 (77.6)	0.002
-Rheumatoid factor	8 (21.1)	2 (25)	0	10 (20.4)	0.737
**AAV features, *n* (%)**					
-General symptoms	21 (55.3)	6 (75)	0	27 (55.1)	0.155
-Respiratory system	20 (52.6)	6 (75)	2 (100)	29 (59.2)	0.24
-Renal	37 (97.4)	2 (25)	0	40 (81.6)	/
-Eye	2 (5.3)	0	0	2 (4.1)	0.76
-ENT	2 (5.3)	0	0	2 (4.1)	0.76
-Cutaneous	11 (28.9)	1 (12.5)	0	12 (24.5)	0.438
-Nervous system	8 (21.1)	1 (12.5)	1 (50)	10 (20.4)	0.504
-Digestive system	4 (10.5)	1 (12.5)	0	5 (10.2)	0.874
-Musculoskeletal system	0	0	0	0	/
-Heart	7 (18.4)	0	0	7 (14.3)	0.34
-c-ANCA/PR3	3 (7.9)	5 (62.5)	0	9 (18.4)	0.001
-p-ANCA/MPO	38 (100)	3 (37.5)	2 (100)	44 (89.8)	/
Other autoimmune diseases, *n* (%)	5 (13.2)	0	0	5 (10.2)	0.48

SS, Sjögren syndrome; AAV, ANCA-associated vasculitis; AITD, autoimmune thyroid disease; ENT, ear-nose-throat; MPA, micropolyangiitis; GPA, granulomatosis with polyangiitis; EGPA, eosinophilic granulomatosis with polyangiitis.

In the 49 patients, 81.6% (40 cases) patients were female. Cases diagnosed as AAV at an average age of 61.5 years, AAV involving the kidney (40 cases), respiratory system (29 cases), ear-nose-throat (ENT) (two cases), nerve system (10 cases), digestive system (five cases), heart (seven cases), eye (two cases). SS involvement: all patients had sicca syndrome, 75.5% (37/49) had gland and kidney involvement, and other systems were affected, such as respiratory system 59.2% (29/49), nervous system 20.4% (10/49), ear, nose and throat 2.1% (2/49). Digestive system 10.2% (5/49), heart 14.3% (7/49).

Laboratory tests showed that the positive rate of anti-SSA/SSB antibody was 77.6% (38/49). The related manifestations of renal tubular acidosis were not mentioned. p-ANCA/MPO positive rate was 89.8% (44/49), c-ANCA/PR3 positive rate was 18.4% (9/49).

Renal pathology: Renal biopsy was performed in 40 cases. Among them, 16 cases (40.0%) showed puci-immune crescentic glomerulonephritis (CGN) with tubulointerstitial nephritis (TIN), 11 cases (27.5%) showed puci-immune CGN, and four cases (10.0%) showed CNG with TIN. Four cases (10.0%), characterized by granulomatous vasculitis, three cases (7.5%), characterized by Focal Segmental Glomerulosclerosis, one case (2.5%) of the glomerulus and renal interstitial fibrosis, one case (2.5%) presented with necrotizing vasculitis.

Treatment progress and outcomes: Most patients (89.8%, 44/49) were treated with corticosteroids, of which 29 (59.2%) were treated with CTX immunosuppressive therapy, six (12.3%) with azathioprine, five (10.2%) with RTX, four (8.2%) with IVIG, and two (4.1%) with hydroxychloroquine, one case (2%) with Leflunomide. Five cases (10.2%) treated with plasma exchange (PE), three cases (6.1%) treated with hemodialysis (HD). Two patients died of acute heart, kidney and respiratory failure due to rapid disease progression. Another five cases could not be classified because of incomplete pathological data. The renal function of other patients was improved or stable compared with baseline.

## 4 Discussion

Anti-neutrophil cytoplasmic antibody-associated vasculitis is a systemic autoimmune disease characterized by oligoclonal immune complex deposition and crescentic glomerulonephritis (9, 10). This case involves an elderly male presenting with hematuria and progressive serum creatinine elevation. Positive p-ANCA/MPO antibodies and renal pathology consistent with focal segmental necrotizing glomerulonephritis led to a diagnosis of MPA according to the 2022 ACR/EULAR criteria (11–13). Concurrently, the patient tested positive for anti-SSA/Ro-52 antibodies, presented with SS symptoms, and had supportive salivary gland biopsy findings. Based on the 2016 criteria, SS was also confirmed (7).

This case raises a core clinical question: Is this an overlap syndrome of AAV and SS, or is it a case of one disease predominating with non-specific antibody positivity for the other? A literature review indicates that ANCA positivity is not extremely rare in connective tissue disease (CTD) patients, with approximately 11% of SS patients testing positive for ANCA. Nishiya et al. (14) suggested that ANCA positivity in SS may merely represent a concomitant phenomenon of B-cell polyclonal activation, lacking independent clinical significance. However, other studies (15) have indicated that ANCA positivity may correlate with vasculitic clinical manifestations and prognosis. The renal pathology in this patient demonstrated active crescent formation or necrosis in approximately 30% of glomeruli, a hallmark feature of AAV rather than the tubulointerstitial nephritis commonly seen in SS (16). Furthermore, the ANCA positivity rate in CTD patients is indeed higher than in the general population (17). Collectively, this case is more consistent with an overlap syndrome representing the coexistence of two distinct diseases.

We further explored the potential mechanisms underlying several distinctive findings in this case. First, why did transient anti-dsDNA antibody positivity occur? Although this antibody is a highly specific marker for systemic lupus erythematosus (SLE) (18, 19). It can also be detected in other CTDs such as SS. During the inflammatory process in SS, the release of nuclear components may induce the production of such antibodies. This case lacked characteristic pathological changes of lupus nephritis, such as diffuse nuclear staining on immunofluorescence. Furthermore, the antibody remained transiently positive, serum complement levels were normal, and SLE could be excluded.

Second, potential causes of thrombocytopenia in the patient. Both the autoimmune diseases themselves (AAV or SS) and the immunosuppressive agent CTX can lead to thrombocytopenia. Given the concomitant leukopenia, bone marrow suppression was the primary consideration; however, bone marrow aspiration results showed no clear abnormalities, leading to a diagnosis of secondary ITP. CTX can cause both bone marrow suppression and antibody-mediated immune destruction of platelets (20). The mechanism of thrombocytopenia in AAV may involve immune-mediated platelet destruction, consumption due to vasculitis itself, and enhanced activation of neutrophil extracellular traps. Multiple autoantibodies present in SS may also attack platelets or cause their destruction via the immune complex pathway (21–24). Although studies have suggested a role for the CD40/CD40L co-stimulatory pathway in ITP (25–30), antiplatelet antibodies were undetectable in nearly half of patients, prompting us to omit this test (31). Given the unclear etiology and associated risks, clinical management opted to discontinue CTX.

Through a literature review of 49 similar cases, we found that patients with this type of overlap syndrome may also develop other autoimmune diseases such as rheumatoid arthritis, anti-glomerular basement membrane disease, and systemic sclerosis (32–34). Although SLE was excluded in this case, the transient presence of multiple autoantibodies indicates a highly active immune system. This suggests a potentially higher risk of developing lymphoma, thymoma, or other plasma cell disorders compared to the general population, underscoring the necessity for long-term follow-up. The literature also indicates that when clinical manifestations are complex and difficult to explain by a single disease, the possibility of an overlap syndrome should be actively considered (35).

This case highlights the importance of recognizing AAV in clinical practice, particularly in the complex setting of concomitant autoimmune diseases, revealing the challenges in achieving accurate diagnosis and developing individualized treatment strategies. The therapeutic goal is to achieve and maintain disease remission while minimizing drug-related complications. The literature supports the view that timely diagnosis and initiation of appropriate immunosuppressive therapy are critical for managing AAV, preventing disease progression, and avoiding organ damage. Treatment typically involves the combination of corticosteroids with immunosuppressive agents, adjusted according to the severity and extent of organ involvement (36). Close clinical and laboratory monitoring serves as the cornerstone for adjusting treatment regimens and optimizing long-term prognosis.

## 5 Conclusion

Through the diagnostic and therapeutic experience of this case and a review of the literature, we recognize that for patients with autoimmune diseases who have complex conditions or poor response to conventional treatments, testing for specific antibodies is crucial for preventing or diagnosing potential serious complications at an early stage. In cases where patients exhibit rapid progression of renal dysfunction, it is crucial to promptly conduct a renal biopsy to confirm the pathological diagnosis. Patients with AAV and SS often present with evident multi-system involvement, and their renal pathology frequently manifests as crescentic nephritis, leading to rapid deterioration of renal function. Once the diagnosis is established, immediate initiation of hormone therapy combined with immunosuppressants is recommended, while plasma exchange should be employed when deemed necessary.

## Data Availability

The original contributions presented in this study are included in this article/supplementary material, further inquiries can be directed to the corresponding authors.
